# Defects in the signaling lipid PI3P may contribute to dendritic spine loss in Alzheimer’s disease

**DOI:** 10.1002/alz.086935

**Published:** 2025-01-03

**Authors:** Pilar Rivero‐Rios, Tunahan Uygun, Garrett D. Chavis, Michael A. Sutton, Lois S. Weisman

**Affiliations:** ^1^ University of Michigan, Ann Arbor, MI USA

## Abstract

**Background:**

Alzheimer’s disease (AD) is the leading cause of dementia worldwide. The recent announcement that lecanemab, a monoclonal antibody targeting amyloid‐b, can slow down cognitive decline in AD is a great step forward in the battle against the disease. However, the modest success achieved in the clinical trial speak to the need for developing additional pharmaceutical approaches to target other key features of AD. Notably, dendritic spine loss correlates more strongly to cognitive decline in AD than amyloid pathology, but the molecular causes remain poorly understood.

**Method:**

We performed immufluorescence studies in cultured primary hippocampal neurons to evaluate endosomal defects and dendritic spine loss.

**Result:**

We discovered that endocytic recycling of cell surface receptors from endosomes to the plasma membrane is key to maintain dendritic spine density in cultured hippocampal neurons. There are two major pathways for endocytic recycling: SNX27‐Retromer and SNX17‐Retriever. Our unpublished data show that both pathways contribute to dendritic spine maintenance by promoting the recycling of different sets of surface proteins at synapses. Moreover, alterations in either pathway result in a loss of dendritic spines.

In addition, we found that the recruitment of SNX17 and SNX27 to synapses is dependent on the signaling lipid phosphatidylinositol‐3‐phosphate (PI3P). Notably, a lipidomic study revealed that PI3P levels are decreased in postmortem brain tissue from AD patients as well as in AD‐related mouse models. Thus, the observed decrease in PI3P levels may contribute to spine loss in AD and act in part by dysregulating endocytic recycling at synapses.

**Conclusion:**

Continued studies of the connections between PI3P, spine density and AD may ultimately open avenues for new therapeutic approaches for this devastating disease.

